# To the Editors of the Medical and Physical Journal

**Published:** 1809-03

**Authors:** 


### *To the Editors of the Medical and Physical Journal.*

GENTLEMEN,

IN a conversation with some young medical friends, who had just left the University, and proposed settling in the country, they expressed a wish that I should point out such works on the different departments of medicine, surgery, midwifery, &c. as would constitute a library of information.

I humbly request any of your Correspondents, who may be better qualified to undertake the task, to favour them with a list through the medium of your useful Journal.

I am, &c.

SENEX.

CRITICAL

